# Radiation and electrostatic resistance for ultra-stable polymer composites reinforced with carbon fibers

**DOI:** 10.1126/sciadv.add6947

**Published:** 2023-03-17

**Authors:** Michal Delkowski, Christopher T.G. Smith, José V. Anguita, S. Ravi P. Silva

**Affiliations:** ^1^ Advanced Technology Institute, Department of Electrical and Electronic Engineering, University of Surrey, Guildford, Surrey GU2 7XH, UK.; ^2^ Airbus Defence and Space GmbH, Claude-Dornier-Strasse, 88090 Immenstaad, Germany.

## Abstract

Future space travel needs ultra-lightweight and robust structural materials that can withstand extreme conditions with multiple entry points to orbit to ensure mission reliability. This is unattainable with current inorganic materials. Ultra-highly stable carbon fiber reinforced polymers (CFRPs) have shown susceptibility to environmental instabilities and electrostatic discharge, thereby limiting the full lightweight potential of CFRP. A more robust and improved CFRP is needed in order to improve space travel and structural engineering further. Here, we address these challenges and present a superlattice nano-barrier–enhanced CFRP with a density of ~3.18 g/cm^3^ that blends within the mechanical properties of the CFRP, thus becoming part of the composite itself. We demonstrate composites with enhanced radiation resistance coupled with electrical conductivity (3.2 × 10^−8^ ohm⋅m), while ensuring ultra-dimensionally stable physical properties even after temperature cycles from 77 to 573 K.

## Teaser

Carbon-fiber composites are enhanced by superlattice nano-barrier for extreme environments and aerospace applications.

## INTRODUCTION

**Fig. 1.**
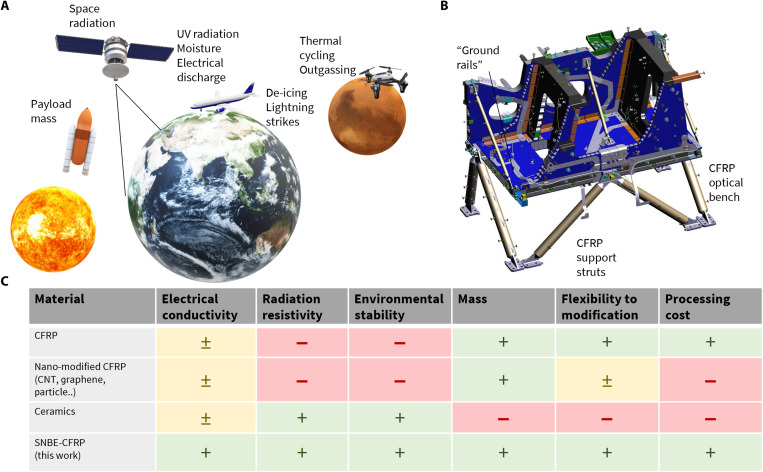
Challenges associated with structural materials selection for space structures. (**A**) Schematic illustration with environmental effects for orbiting satellites, aircrafts, and planetary science missions. (**B**) Image of the Sentinel-5 optical support carbon fiber reinforced polymer (CFRP) structure. (**C**) Comparison of simplified selection matrix for structural satellite components with superlattice nano-barrier–enhanced (SNBE)–CFRP (this work).

For many space missions, highly stable, lightweight, and functional structures are essential to support precision apertures and finely calibrated instruments on satellites (*1*–*4*). Stable structures, such as optical benches, telescope tubes, parabolic reflectors, rovers, and lander structures, are required for precision optics, radar, and scientific instruments for various deep-space, Earth, Observation, Navigation and Science (ENS), and exploration missions (*5*–*11*). It is imperative that these structural components maintain the highest performance under extreme test, launch, and space conditions to ensure mission viability (*12*–*14*). The table in Fig. 1 shows a qualitative comparison of materials commonly considered for structural components, including recent innovations. Most advanced inorganic space materials such as silicon carbide (SiC), silicon nitride (Si_3_N_4_) or Zerodur have high robustness and low coefficient of thermal expansion (CTE) [around 2.2 to 5 parts per million (*15*–*17*)], thus offering some degree of dimensional stability. However, this strength introduces fragility and incurs high weight and cost, while restricting modification after fabrication. The NIRSpec (near-infrared spectrograph) instrument onboard the James Webb Space Telescope experienced these issues with its SiC optical bench exceeding the costs and manufacturing schedule (*18*, *19*), while Zerodur showed radiation-induced compaction on the Semi-Conductor Inter Satellite Link Experiment mirror (the first European optical communication terminal in orbit), thereby leading to deformation (*20*). These materials are also good electrical insulators with an electrical resistivity around 10^8^ ohm⋅m or higher (*15*–*17*). Attempts to enhance these properties, for example, by doping or changing particle concentration and processing steps, have shown to ultimately reduce the mechanical performance in single-crystal materials (*15*–*17*, *20*).

In comparison, lightweight carbon fiber reinforced polymer (CFRP) can be designed to feature near-zero CTE, ensuring dimensional stability at nearly one-third of the weight density (*21*, *22*). This allows the construction of challenging architectures, for example, the rotor blades and legs of the Mars Ingenuity helicopter, with the whole structure weighing only approximately 1.8 kg (*23*). However, the mechanical integrity of CFRP can be hampered by radiation damage of the fiber/matrix interface, which leads to microcracking and loss of mechanical performance (*24*–*26*). Furthermore, radiation flow (both of ions and electrons) can cause electrostatic charging of CFRP. Carbon fibers are good conductors along the length of the fiber, but the resin matrix is an isolator leading to anisotropic conductivity in CFRP. Commonly seen in satellites, CFRP structural parts are connected through a network of the so-called “ground rails” usually implemented as aluminum strips that interconnect various satellite units (Sentinel-5 optical support structure; Fig. 1B). This restricts the full lightweight potential of CFRP and often introduces additional connections or adhesive joints that result in the outgassing of volatile organic compounds (VOCs). Outgassing of VOCs has been shown to lead to clouding of sensitive optical instruments, threatening mission failure (*27*–*29*). Electrostatic shielding is also used in aircraft to prevent lightning strikes (*30*, *31*) or terrestrial applications such as automotive to ensure electrostatic balance (*32*). CFRP electrical properties can be enhanced using carbon nanotubes, doping, etc., but not without drawbacks to other properties (*33*–*36*). Designs for the Rosetta lander structure, launched in 2004, used chromated aluminum-alloy foils, at the expense of reduced structural properties and higher instrument mass (with added mechanical joints) (*24*). Thin-film solutions, such as that proposed for the camera onboard National Aeronautics and Space Administration’s (NASA’s) Mars Observer mission, which proposed an 18-μm-thick, Ni, Au, and In-Sn/In-Bi layer, were ultimately rejected for stable structures as these increased the overall CTE and instrument mass and affected the mechanical performance of CFRP (*24*). Furthermore, a white silicate attempted on the Deep Space Climate Observatory NASA’s mission was also rejected due to fragile behavior, contamination, and electrostatic charging issues (*37*). Recent developments have shown that using a superlattice nano-barrier can provide protection from moisture and outgassing (CVCM = 0.00%) while not affecting the overall CTE of the CFRP and enabling a tandem platform to adhere further layers to (*3*–*4*, *14*). Furthermore, the nano-barrier–protected CFRP has been shown to increase the robustness, wear, and crack resistivity of composite materials (*4*).

This article presents a mechanically coupled, superlattice nano-barrier–enhanced CFRP (SNBE-CFRP) that substantially improves the radiation and environmental resilience of composites by encapsulating the fiber/matrix interfaces. This ensures stable mechanical properties and lightweight designs with outgassing-free performance. The stress-matched surface and amorphous dense barrier structure allows reducing the stresses and contamination at the surface, resulting in strong adhesion integrated as part of the original CFRP layer and mechanical robustness, even after irradiation and thermal vacuum cycles. The mechanically integrated nano-barrier is thin (<1 μm with structure described later) and shows no evidence of damage after exposure to simulated space environment conditions. A plasma-enhanced chemical vapor deposition (PECVD) system is used to deposit the superlattice nano-barrier coating, which is capable of encapsulating complex, three-dimensional (3D) structures at room temperature. Furthermore, a topmost ultrathin (50 to 100 nm) electrically conductive layer can be selectively deposited and mechanically matched to provide electrical CFRP grounding, thereby avoiding the need for additional processing and addition of heavy and risky metallic joining around the satellite structures.

## RESULTS

**Fig. 2.**
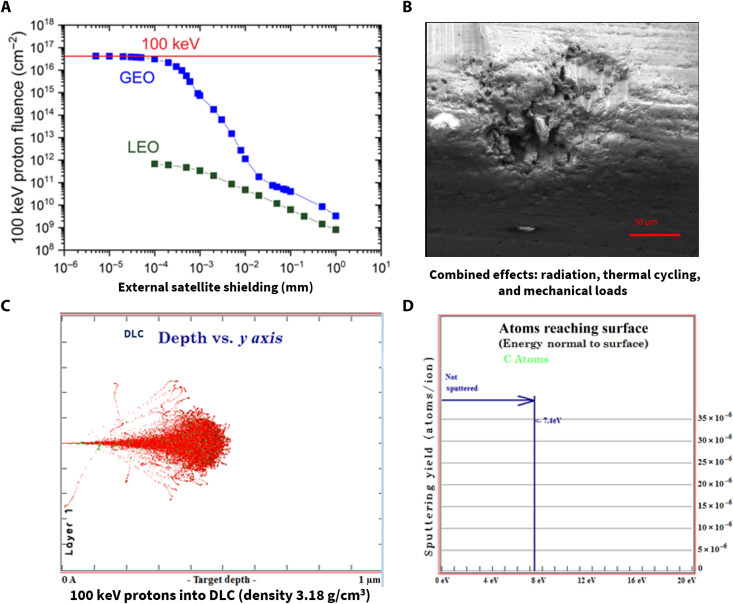
Space radiation and modeling of environmental barrier shielding. (**A**) Modeled (details in Materials and Methods and tables S1 to S3) 100-keV proton fluence as a function of external satellite shielding thickness for low-Earth orbit (LEO) and geo-stationary orbit (GEO), 10-year mission length (e.g., Sentinel-5 and Sentinel-4 satellites). (**B**) Image showing degradation of unprotected CFRP (matrix/fiber interface) after 100-keV proton irradiation for the GEO environment from the defined mission profile (A) with further microcracking as a result of combined thermal cycling and mechanical loads (Fig. 4A). (**C**) Modeling showing the capability of the thin diamond-like carbon (DLC) layer (500 nm) to fully shield against radiation as a function of penetration depth, i.e., DLC absorbing radiation for modeled GEO worst-case conditions (A). (**D**) Atoms absorbed by the DLC barrier layer with less than 8 eV reaching the surface, thereby avoiding scattering at the fiber/matrix interface of CFRP.

A common method for radiation protection in space applications is material shielding with aluminum. This requires both external and internal shielding to protect from trapped protons and electrons, as found in the Van Allen belts (*12*–*14*). In essence, charged particles including protons with energies ranging from 0.1 to 400 MeV are captured by the strong magnetic field of the planet forming the radiation belts, typically an inner belt with an altitude of about 6000 and 12,000 km and an outer belt around 25,000 and 45,000 km, thus directly affecting satellites in orbit. However, aluminum shielding is ductile, not thermally stable or fully conformal, and therefore usually undesired for stable structures, also contributing substantially to satellite mass and costs (*24*). Figure 2A shows modeled maximum fluence of 100-keV protons reaching the structures on Copernicus missions for the geo-stationary orbit (GEO) and the low-Earth orbit (LEO) (e.g., Sentinel-4 and Sentinel-5, respectively) with data obtained from the Space Environment, Effects and Education System (SPENVIS; details are given in tables S1 to S3). The high proton fluence (at lower energy) that scatters on the surface causes a major degradation of the resin matrix phase for stable CFRPs, as fibers inhibit bulk degradation of the composite. This was shown by the European Space Agency (ESA) and NASA (*24*), and demonstrated on test CFRP composites (Fig. 2B) where degradation of the fiber/matrix interface and microcracking is encountered and further propagates as a result of combined environmental effects such as thermal cycling and mechanical loads. This type of defect whether in operational structures or space structures will ultimately cause these to fail with severe costs, also inducing very high operational limitations.

**Fig. 3.**
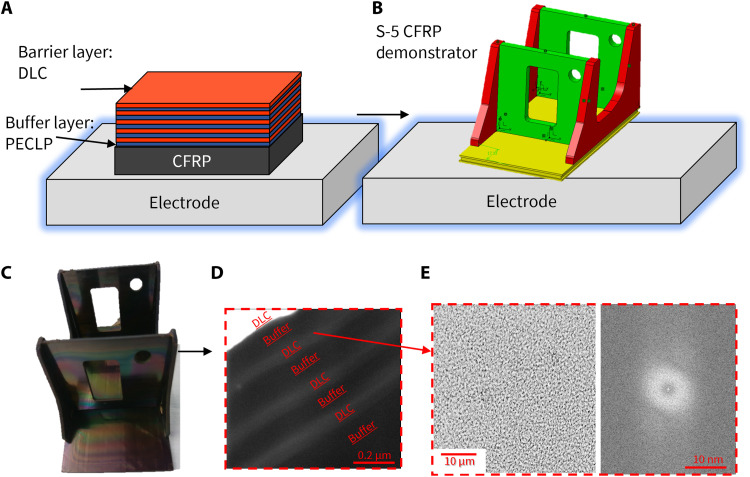
Environmental enhancement of CFRP. (**A**) Schematic illustration showing SNBE-CFRP with the multilayer barrier consisting of DLC barrier and PECLP buffer layers deposited in one process run. (**B**) Model of the Sentinel-5 optical module (Fig. 1B) before manufacturing showing the deposition setup with the connected structure to the radio frequency electrode. (**C**) Conformally coated Sentinel-5 demonstrator model. (**D**) Scanning electron microscope (SEM) cross section showing nano-barrier layers after thermal shock and adhesion tape testing (described in Materials and Methods). (**E**) DLC image from transmission electron microscopy analysis revealing no evidence of clustering or ordering in the barrier layer with a Fourier diffractogram of the amorphous carbon ring of the DLC barrier.

Physical barriers that absorb unwanted radiation and increase environmental stability can be made of highly amorphous materials, as shown for solar cells using ion-beam doping (*38*, *39*). However, these are typically achieved only for flat and uniform samples, with deposition processes at elevated temperatures. Furthermore, the performance of metallic- and ceramic-based materials degrade rapidly as the deposition temperature is reduced to values near 100°C or lower, as required for deposition onto CFRP and sensitive polymers (*40*, *41*). Consequently, here, a PECVD process was selected for its ability to conformally coat complex 3D structures. Diamond-like carbon (DLC) is used as the physical barrier material, using a room-temperature deposition method. Figure 3E shows that the DLC deposited at room temperature is a dense and sp^3^-rich (sp^2^/sp^3^ ratio 8%) amorphous structure of a-C:H, as measured by electron energy-loss spectroscopy (EELS) (fig. S1) using a transmission electron microscope (TEM). Furthermore, TEM and Fourier transform analysis of the images demonstrate no evidence of pinholes and no evidence of clustering or ordering, but only of a dense barrier layer capable of encapsulating from external environmental effects. To overcome the considerable challenges posed by coating CFRP directly, a flexible plasma-enhanced poly-(p-xylylene) (PECLP) buffer layer is used, which has been shown to allow the production of thermomechanical stable structures (*3*, *4*). Organic polymers such as PECLP are typically sensitive to proton radiation bombardment, resulting in a combination of further cross-linking, chain scission, and ultimately depolymerization (*42*, *43*). This can result in poor adhesion to the substrate, leading to blistering on the surface if left unprotected.

Figure 3B presents a computer model of the Sentinel-5 instrument support structure demonstrator (instrument depicted in Fig. 1B), showing a coated CFRP mock-up on Fig. 3C. The structure of the coating is shown schematically in Fig. 3A with a continuously growing process during conformal deposition of nano-barrier coating. Figure 3D shows a cross section of the coating after deposition on a complex CFRP surface that is highly structurally featured, demonstrating that the desired coating is conformal.

To ensure adequate protection, a sufficient thickness of non–radiation-sensitive shielding material is required. Having modeled the radiation penetration of deposited DLC with a density of ~3.18 g/cm^3^, it was established that the DLC barrier layer is capable of shielding CFRP against scattered radiation when it is only as thin as 500 nm (Fig. 2C). The DLC superlattice encapsulates the CFRP and PECLP networks, each of which is a weak point. This suggests that the dose of 100-keV protons and a fluence of 4.18 × 10^16^ cm^−2^ representing the worst case for GEO derived from Copernicus missions (Fig. 2A) can be effectively absorbed by the DLC superlattice structure, thereby preventing scattered atoms from reaching the CFRP surface (Fig. 2D). The DLC superlattice is also highly amorphous and very thin, and so it does not affect the bulk properties of CFRP.

**Fig. 4.**
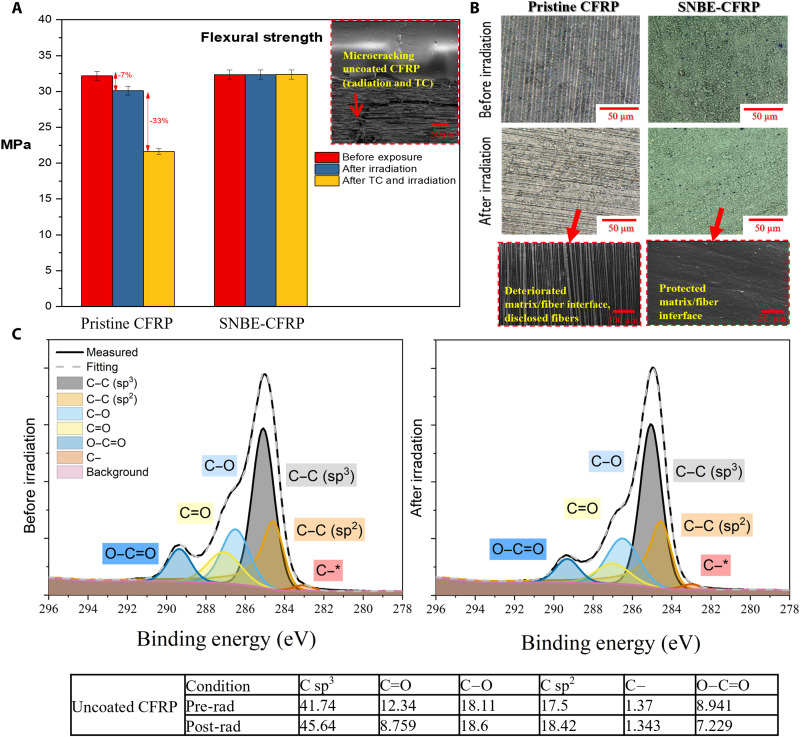
Environmental testing and analysis of CFRP and SNBE-CFRP. (**A**) Mechanical bending test results on CFRP and SNBE-CFRP (minimum of 20 samples for each configuration; a summary is given in table S4), before environmental exposure, after proton irradiation (dose in Fig 2A and table S1 to S3), and after proton irradiation and thermal vacuum cycling (TC) between 233.15 and 333.15 K (inset: SEM image with generated microcracking as a result of thermal cycling and mechanical loads, which was first initiated by proton irradiation that removed the resin matrix). (**B**) Optical and SEM images showing the deteriorated unprotected CFRP resin matrix as a result of proton irradiation, and fully protected SNBE-CFRP without a sign of deterioration (the image is taken after additional thermal shock and adhesion tape tests described in Materials and Methods). (**C**) X-ray photoelectron spectroscopy (XPS) analysis before and after irradiation on unprotected CFRP showing chain scission, removal of resin matrix, and fiber disclosure (increased carbon content: sp^3^ and sp^2^); these results are consistent with previous SEM and optical observations (B) (XPS for SNBE-CFRP is shown in fig. S2).

As a result of the SNBE-CFRP structure, the fiber/matrix interface and interphase elements such as mechanical interlocking are encapsulated and protected, thereby prepared for further environmental testing. Figure 4A shows the mechanical test results from three-point bending tests performed on SNBE-CFRPs and unprotected CFRPs, after and before simulated space conditions. A proton irradiation dose of 100 keV was applied (Fig. 2 and fig. S1) across multiple CFRP samples, reaching 4.18 × 10^16^ cm^−2^, and thus representing a GEO worst-case scenario for a 10-year mission length. The results show that the flexural strength is decreased by 7 and 3.5% for pristine CFRPs (Unidirectional (UD) 90° and 0° layup, respectively; this is perpendicular and in parallel to the fiber) after proton irradiation, whereas SNBE-CFRP ensures stable mechanical performance. These values for unprotected CFRPs are consistent with previously reported results by space agencies (*24*). This deterioration is associated with scattered atoms and subsurface degradation of the matrix resin phase, which further leads to microcracking and CTE changes of the CFRP, both of which are crucial physical parameters for advanced structures. Proton irradiation shows signs of first removing the resin matrix and then CFRP becomes more prone to environmental damage. Consequently, subsequent thermal cycling and mechanical loads result in enhanced microcracking (SEM in Figs. 4A and 2B). Figure 4B demonstrates this behavior, where optical images are taken before and after irradiation on pristine CFRPs and show surface morphological modifications. A scanning electron microscopy (SEM) image depicts (Fig. 4B, bottom) clear deterioration of the subsurface fiber/matrix interphase (resin removal), thereby exposing the fibers out of the composite surface. In contrast, the superlattice nano-barrier protects the CFRP, and because it is ultrathin (<1.5 μm) and mechanically coupled, it does not hinder mechanical properties of the composite (~32.25 and ~528.7 MPa, for UD90° and UD0° CFRPs, respectively). The x-ray photoelectron spectroscopy (XPS) analysis before and after irradiation reveals damage of the resin matrix to unprotected CFRP, which is consistent with previously reported results (*24*–*26*). These results suggest a chain scission and separation of molecular products from the resin network leading to its recombination and chain mobility. It is shown on the XPS that carbon content (sp^3^ and sp^2^ ratio) is increased, which means that irradiation breaks down the chemical bonds of resin leading to microcracking (visible in Figs. 2B and 4A). This analysis is consistent with earlier observations from optical microscopy and SEM images (Fig. 4A). The SNBE-CFRPs showed no signs of deterioration (the XPS is given in fig. S2).

Aside from radiation, satellites operate under temperature extremes. It has been shown that crack density is further encountered as a result of thermal cycles (*3*–*5*, *24*). This is shown in Fig. 4A, where unprotected UD90° CFRPs decrease flexural strength by 33% (15% decrease for UD0° CFRPs) after the thermal-vacuum cycles (100 cycles), between 233.15 and 333.15 K (e.g., qualification temperature range of Sentinel-5 support structure). In contrast, the SNBE-CFRP still exhibits stable mechanical properties even after thermal-vacuum cycling and irradiation exposure. Furthermore, the SNBE-CFRP has been further exposed to temperature cycles from 77 to 573 K to simulate temperature fluctuations greater than those experienced on thermally stable optical benches. This temperature range did not cause evidence of delamination, even after adhesion tape tests (e.g., Fig. 4B and fig. S3), thereby demonstrating environmentally stable SNBE-CFRP structure.

**Fig. 5.**
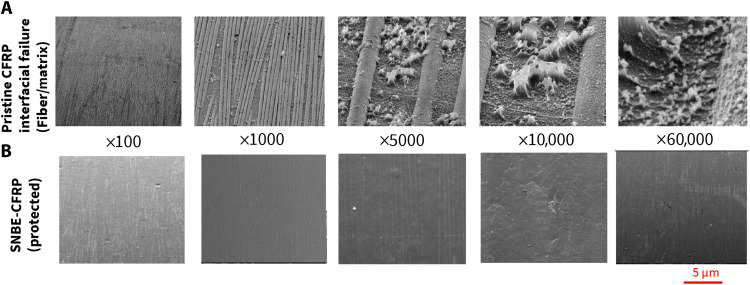
SEM imaging during ion/electron exposure on CFRP and SNBE-CFRP. (**A**) Images (magnification, ×100 to ×60,000) showing interfacial failure at the resin matrix/fiber interface for uncoated CFRP as a result of ion/electron exposure (100 keV). (**B**) SNBE-CFRP with a stable and protected structure via a superlattice nano-barrier during the ion/electron exposure up to 100 and 200 keV (images with magnification, ×100 to ×60,000).

Unprotected CFRPs and SNBE-CFRPs were also evaluated against electron and ion exposures with in-situ SEM inspection. Previous studies from NASA and ESA reported that fiber/matrix interfaces deteriorated, leading to increased microdamage of CFRP (*3*–*5*, *24*). This is shown in Fig. 5A, where our pristine CFRP begins to lose the interfacial adhesion between fiber and resin matrix during the ion/electron irradiation (100 keV). This further breaks down the resin and causes microdamage, which is visible on Fig. 5A. A separation between fibers and resin can be distinctly observed on Fig. 5A with a magnification of ×5,000 to ×60,000. In contrast, the nano-barrier creates a single-composite entity with CFRP, protecting it against such failures. Figure 5B shows in situ SEM imaging during ion/electron exposure (100 keV) with undamaged SNBE-CFRP.

As a result of the reduced surface stresses, enhanced environmental protection, and interfacial adhesion at the top of SNBE-CFRP, it is now possible to couple further layers to adapt additional functionalities, for example, optical or electrical requirements. With this, an enhancement of electrical resistivity to 3.2 × 10^−8^ ohm⋅m is demonstrated (from pristine CFRP, with resistivity >1 × 10^6^ ohm⋅m). This was made possible by using the superlattice nano-barrier as a platform for the deposition of an ultrathin (50 to 100 nm) capping layer of aluminum. This, in turn, raises the SNBE-CFRP conductivity to avoid electrostatic discharge, thereby easing the structural design and allowing the full lightweight potential of CFRP to be taken advantage of when compared to the current grounding solutions used by industry (e.g., space “ground rails”; Fig. 1B).

## DISCUSSION

To summarize, a technology is presented for the design and manufacture of ultra-stable composites with enhanced radiation and electrostatic resilience by the application of a mechanically robust multilayer barrier coating, which is deposited at room temperature and encapsulates the matrix/fiber interfaces of composites. This is made possible by a highly dense superlattice structure, which is coupled to the CFRP, creating a single composite entity. The resulting composite (here named SNBE-CFRP) shows considerably improved space environment stability and functionality over the conventional space-qualified CFRP, resulting in an advanced material that features ultrahigh performance and lightweight potential for instrument applications in space. The nano-barrier achieves this protection even for thickness values below 1 μm, thereby not affecting the overall bulk CFRP properties. The coating stability (under harsh space conditions) allows the deposition of capping layers to obtain further functionalities, for example, electrical conductivity, thereby lowering the instrument mass and showing important improvements over the conventional method of electrical grounding of space structures using metallic straps. This provides evidence of the applicability of the SNBE-CFRP for advanced space applications where ultrahigh and stable properties are required under extreme environmental conditions. Technologies such as this often find applications in a wide field of engineering branches such as photonics, electronics, and photovoltaics, thereby benefiting innovation and industry generally.

## MATERIALS AND METHODS

### CFRP manufacturing

CFRP composites were manufactured using hand layup and fiber tow placement facility capable of ultrahigh-modulus fiber processing for aerospace applications. The ultrahigh-modulus PITCH “Granoc” carbon fibers and fabrics (>700 GPa) with thermoset epoxy matrix systems were used. Two epoxy systems, an epoxy-anhydride (bisphenol A diglycidylether epoxy resin with a methyl nadic anhydride hardener) and an epoxy-amine [bisphenol A diglycidylether epoxy resin with a dimethyl-methylene bis(cyclohexylamine) curing agent], were used to produce CFRP composite samples for testing. These allowed a close-to-zero CTE to be achieved and, after applying the SNBE, also a close-to-zero CME (in-plane and out-of-plane), resulting in ultrahigh stable composites. A vacuum infusion process was used, thereby ensuring a minimum of 60% (±1%) of fiber volume content (FVC). The FVC and differential scanning calorimetry were performed as a standard quality control measurement. A minimum of 125°C of the glass transition temperature was ensured. The measurements were taken across the manufactured plates from which the samples were cut and provided with strictly controlled properties. During the environmental testing, the composites with quasi-isotropic (±45°/90°/0°)_s_ and UD (90°)_16_ and UD (0°)_16_ layups were tested. Both exhibited the same behavior. The UD samples were further used to investigate and assess the degradation impact on mechanical performance after irradiation (in-plane and out-of-plane) for pristine and SNBE CFRPs. The samples (dimension, etc.) were manufactured in accordance with the further provided test standards.

### Deposition

SNBE-CFRP coating layers were deposited under vacuum (without interruption) and at room temperature, using a custom-built PECVD coating system where CFRP substrates were directly connected to the electrically driven electrode with the radio frequency power supply (13.56 MHz), thereby creating a virtual electrode (Fig. 3). The buffer layer was the PECLP and the hard inorganic barrier layers were plasma-deposited DLC. All layers were deposited continuously, without interrupting the plasma. A poly(p-xylylene) dimer di-para-xylylene {[2.2]Paracyclophane (97%), Synonom: Tricyclo[8.2.2.24,7] hexadeca-4,6,10,12,13,15-hexaene} was pyrolyzed at 650°C to create the poly(p-xylylene) monomer and then introduced into an active plasma within the PECVD chamber to form the PECLP (C16H16, detail analysis) (*4*). A hydrogen/acetylene mixture was incorporated into the system, where hydrogen acted as a carrier gas, carrying the acetylene precursor to provide the DLC layer deposition. The initial PECLP layer was 500 nm thick and the DLC layers were always 100 to 200 nm thick. The second and subsequent PECLP coating layer(s) that were deposited on the top of the initial PECLP:DLC superlattice were reduced to 100 nm.

In total, a PECLP:DLC superlattice with eight layers [as stated: (i) PECLP: 500 nm, (ii) DLC: 100 to 200 nm, (iii) PECLP: 100 nm, (iv) DLC: 100 to 200 nm, (v) PECLP: 100 nm, (vi) DLC: 100 to 200 nm, (vii) PECLP: 100 nm, and (viii) DLC: 100 to 200 nm] was constructed to provide the required DLC thickness (Fig. 2) for encapsulation and environmental shielding. A minimum of four layers of PECLP:DLC superlattice structure was found to provide protection from moisture and outgassing (*3*). The PECLP coating was deposited to mitigate surface stresses of the substrates, increase the adhesion, and provide physical environmental barrier. The top capping layer was aluminum (50 to 100 nm thick) to impart electrical functionality. This thin layer is semi-flexible and has higher CTE, and therefore, it could be strongly coupled on top of SNBE with no adhesion issues.

### Modeling, environmental testing, and characterization

The radiation environment was modeled and characterized using Airbus tools and space heritage for different orbit setups (fig. S1). In addition to MEO and GEO, three heliosynchronous LEOs were investigated. The L5 orbit was defined as near-Earth interplanetary orbit. All data were obtained in SPENVIS based on the inputs for the orbit parameters defined (see fig. S1). For all orbits, the mission duration was 10 years with launch dates of 7 years maximum and 3 years minimum. This corresponded to one year of maximum solar activity, with an additional 20 orbits considered in the analysis. The external shielding material was aluminum evaluated from commonly used materials (i.e., housing, MLI, and composites) based on real mission architectures, thereby defining the worst-case (highest) fluence scenarios. The SRIM (Stopping and Range of Ions in Matter) Monte Carlo simulation model was used to assess DLC capability for radiation shielding (Fig. 2), as the PECLP was used as a buffer layer to mitigate thermomechanical stresses. The three-point bending tests (Fig. 4A) were used to measure flexural properties of SNBE-CFRPs and pristine CFRPs. The tests were carried out in a mechanical Zwick machine 1474 in accordance with DIN EN ISO 14125 with the following parameters:

Load cell 10 kN

Jig distance for UD0°: 80 mm

Jig distance for UD90°: 40 mm

Cross head velocity for UD0°: 2 mm/min

Cross head velocity for UD90°: 0.5 mm/min

A minimum of 20 samples per configuration were tested and manufactured in accordance with the standard, which had the following dimensions: 100 mm by 15 mm by 2 mm and 60 mm by 15 mm by 2 mm.

The irradiation tests were carried out at the Surrey Ion Beam Centre (IBC) where determined dose profiles were applied and achieved (Fig. 2A and tables S2 and S3) after approximately 4 days. The dose rate was approximately 1.09 × 10^12^ H/cm^2^ per second, reaching a total of 4.18 × 10^16^ H/cm^2^ representing the GEO worst-case scenario (Fig. 2A and tables S2 and S3). The tests were carried out using the implantation and irradiation facility, which can achieve 2 keV to 4 MeV (up to 10 mA) and heavy ions up to 10 MeV. The IBC is the national U.K. facility and enables hot (700°C) or cold (~10 K) testing. By ensuring controlled handling, the samples were mounted to the special holder and further to the sample chamber in a class 100 clean room. The tests were carried out under vacuum with a set temperature limit below 60°C not to degrade CFRP materials. This was controlled in situ (including blank calibration tests). In addition, FEI Nova NanoLab 600 was used for electron and ion exposure testing and in situ sample imaging with a minimum fluence of 100 keV. A field emission scanning TEM, operated at 200 keV, with a nominal image resolution of 1.7 Å, fitted with a Gatan Enfina electron energy-loss spectrometer was used for EELS analysis. The sp^3^ fraction was used to determine the density of the DLC material and the choice of the refractive index; the procedure of sp^2^ calculation and the derivation of the density are given in the literature (*44*). Radiation testing was performed in accordance with ISO15856 and ECSS-Q-ST-70-06C, including appropriate composite preparation, handling, and conditioning (before/after the tests) to avoid any oxidation effects, respectively.

Thermal vacuum cycling in the range −40° to 60°C was performed in a thermal vacuum chamber with a typical heating rate of 10°C/min. The tests were carried out according to the ECSS-Q-ST-70-04C standard with cycling commencing after reaching the working vacuum of 1 × 10^−5^ Pa. The final inspection and testing at ambient and relative humidity (RH) (55±) conditions were carried out. The tests were realized at the space thermal-vacuum laboratories in Germany. Furthermore, thermal-vacuum cycling tests were performed several times during development by exposing substrates up to 100 cycles ranging from 77 to 573 K.

The adhesion tape test has been carried out on all SNBE-CFRP substrates before and after particular tests to verify coating robustness. The test is in accordance with both ISO9211-4 and ECSS-Q-ST-70-17C standards. The tape used was transparent with a minimum adhesive strength of 9.8 N per 25-mm width (measured on a steel reference). At least 5 cm of pressure-sensitive tape was attached to the surface with pressure applied to ensure that it adhered well to the surface. The tape was then pulled at right angles at a rate of approximately 1 cm s^−1^. Any remains of coating were inspected on the tape, if any. All components were tested and passed the adhesion tape test after undergoing thermal cycling in LN_2_. Dektak profilometer, SEM (using FEI Quanta), TEM, and in situ quartz oscillator measurements were used to inspect and measure the SNBE-CFRP thickness. The EELS/TEM measurements were used to assess the DLC barrier structure. The XPS spectra (Fig. 4C and fig. S2) were obtained to analyze samples before and after radiation. Optical measurements were done using a Keyence VHX-500 microscope. The electrical resistivity was measured using the four-point probe technique according to the ECSS-E-ST-20-06C standard (spacecraft charging).
