# Supplementary material for: Radiation and electrostatic resistance for ultra-stable polymer composites reinforced with carbon fibers
Source: Sci Adv. 2023 Mar 17;9(11):eadd6947. doi: 10.1126/sciadv.add6947 (PMC10022895; doi:10.1126/sciadv.add6947)
Supplement: Supplementary file 1 — Figs. S1 to S3 Tables S1 to S4 [file sciadv.add6947_sm.pdf]

Science Advances
AAAS

Supplementary Materials for

Radiation and electrostatic resistance for ultra-stable polymer composites reinforced with carbon fibers

Michal Delkowski *et al.*

Corresponding author: S. Ravi P. Silva, s.silva@surrey.ac.uk

Sci. Adv. **9**, eadd6947 (2023)
DOI: 10.1126/sciadv.add6947

This PDF file includes:

Figs. S1 to S3
Tables S1 to S4

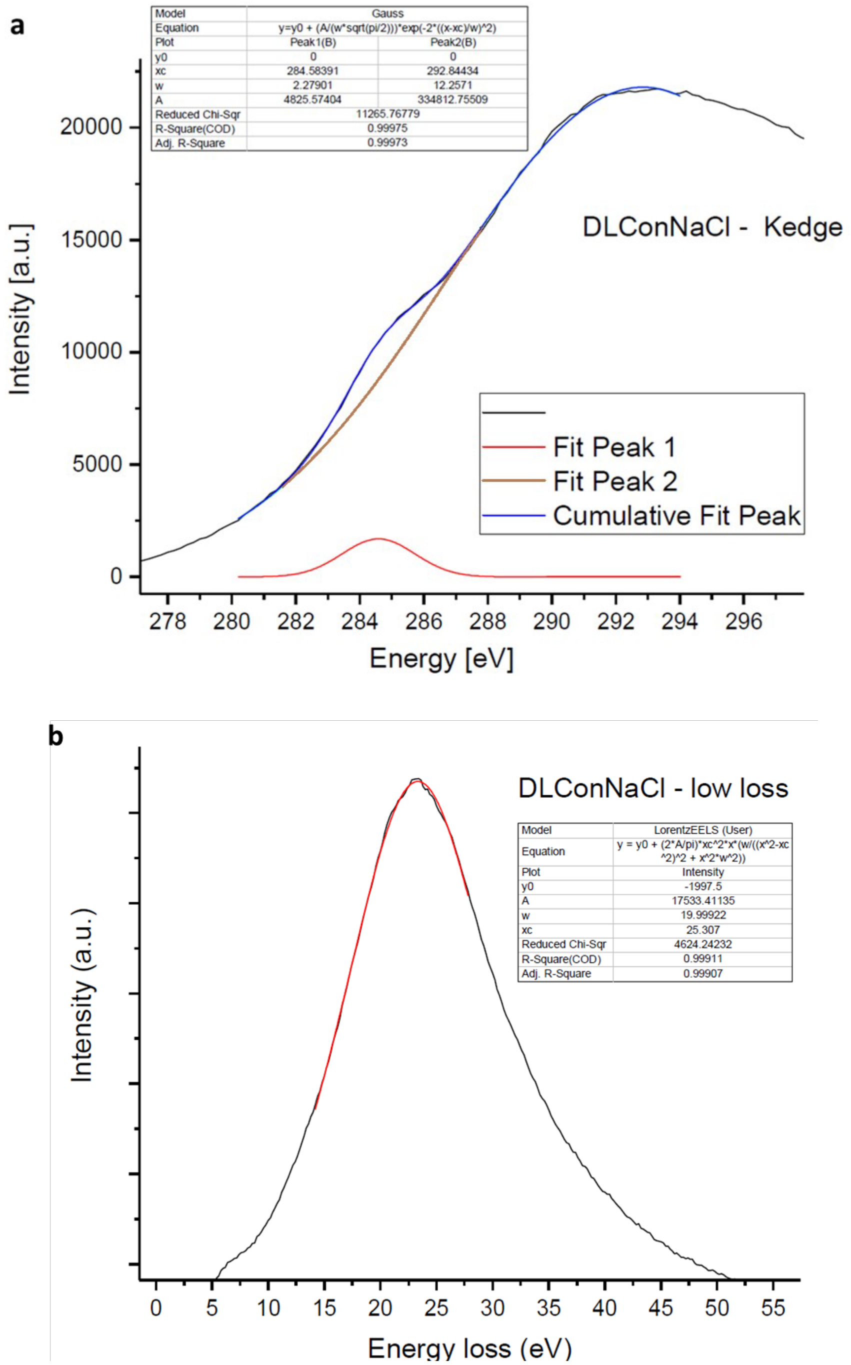

Fig. S1. EEL spectra collected from the beam in the absence of the specimen.
Spectra including the low-loss peak determining the plasmon energy peak position and density derived from the procedure described (*44*)**.**

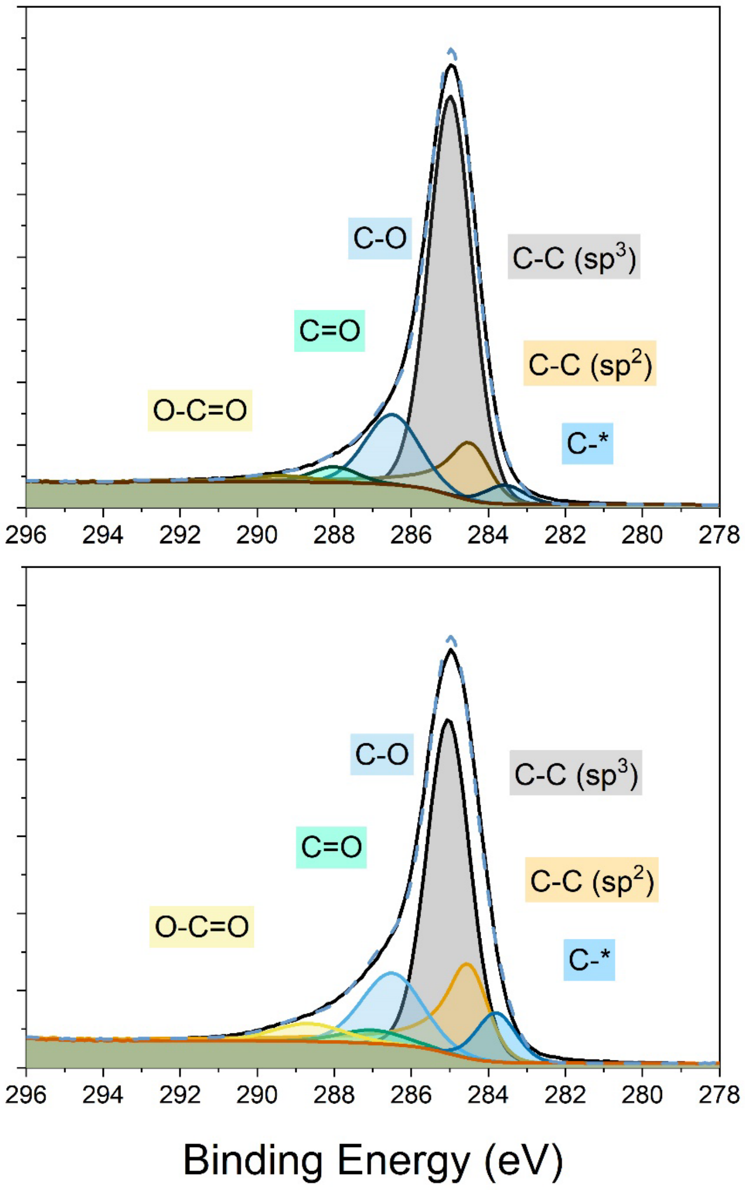

	Condition	C sp3	C=O	C-O	C sp2	C-	O-C=O
SNBE-CFRP	Pre Rad	68.1	2.835	15.03	9.604	2.908	1.525
	Post Rad	53.89	3.227	16.15	14.89	7.202	4.644

Fig. S2. X-ray photoelectron spectroscopy (XPS) analysis pre and post irradiation on SNBE-CFRP.

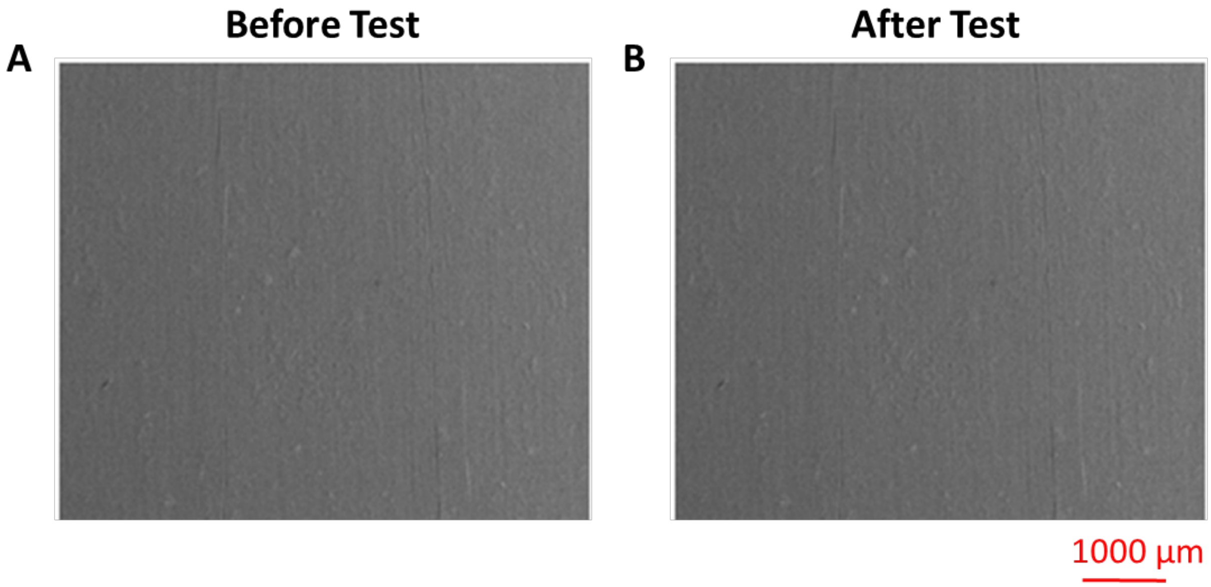

Fig. S3. SNBE-CFRP before and after adhesion tape testing.
(A) SNBE-CFRP before thermal-shock (down to 77k) and adhesion tape testing
(B) SNBE-CFRP after thermal-shock (down to 77k) and adhesion tape testing without sign of delamination

Table S1. Detailed data for orbits investigated.

Orbit	Perigee altitude (km):	Apogee altitude (km):	Inclination (deg):	Orbit Period (min):	Orbit type:
LEO1	725	725	98.29	99	heliosynchronous
MEO	20000	20000	98	710	general
GEO	35870	35870	0	1436	geostationary
	**distance from sun (a.u.)**				
L5	1				near-earth interplanetary

Orbital parameters used to model radiation environment (incl. worst-case scenario) to be encountered by internal instrument space structures.

Table S2. Detailed data for LEO1 orbits investigated.

The fluence of protons as a function of the shielding determined and applied for internal structures at LEO1 (e.g. Sentinel-5)

External absorber thickness (mm)	Fluence [cm^{-2}], 0.1 MeV Protons LEO1
1.00E-004	6.764E+011
2.00E-004	6.100E+011
5.00E-004	4.696E+011
1.00E-003	3.388E+011
2.00E-003	2.045E+011
5.00E-003	8.562E+010
1.00E-002	4.755E+010
2.00E-002	2.660E+010
5.00E-002	1.173E+010
1.00E-001	6.226E+009
2.00E-001	3.212E+009
5.00E-001	1.436E+009
1.00E+000	8.016E+008

Table S3. Detailed data for GEO orbits investigated.

The fluence of protons as a function of the shielding determined and applied for internal structures at GEO (e.g. Sentinel-4).

External absorber thickness (mm)	Fluence [cm^{-2}], 0.1 MeV Protons GEO
5.00E-06	4.18E+16
1.00E-05	4.10E+16
2.00E-05	3.97E+16
3.00E-05	3.84E+16
4.00E-05	3.72E+16
5.00E-05	3.60E+16
1.00E-04	3.09E+16
2.00E-04	2.15E+16
3.00E-04	1.43E+16
4.00E-04	9.63E+15
5.00E-04	5.60E+15
6.00E-04	3.05E+15
9.00E-04	9.13E+14
1.00E-03	7.42E+14
2.00E-03	1.78E+14
3.00E-03	6.23E+13
5.00E-03	1.49E+13
8.00E-03	2.72E+12
1.00E-02	1.14E+12
2.00E-02	1.81E+11
4.00E-02	7.48E+10
5.00E-02	6.46E+10
7.00E-02	5.21E+10
8.00E-02	4.75E+10
1.00E-01	4.02E+10
5.00E-01	8.55E+09
1.00E+00	3.28E+09

Table S4. Summary of mechanical bending test results on CFRPs and SNBE-CFRPs. Mechanical bending test results (in MPa) for CFRP (UD0° and UD90°) and SNBE-CFRP (UD0° and UD90°), before environmental exposure, after proton irradiation (dose in Fig 2A, Table S1 S3), after proton irradiation and thermal vacuum-cycling (TC) between 233.15 and 333.15 K

Material	Before Exposure	After Irradiation	After Irradiation & TC
CFRP UD90°	32.171	30.111	21.612
SNBE-CFRP UD90°	32.352	32.374	32.381
CFRP UD0°	525.944	508.932	447.052
SNBE-CFRP UD0°	528.723	528.814	529.213
